# Supplementary material for: Genome-Wide Identification, Characterisation, and Evolution of the Transcription Factor WRKY in Grapevine (Vitis vinifera): New View and Update
Source: Int J Mol Sci. 2024 Jun 5;25(11):6241. doi: 10.3390/ijms25116241 (PMC11172563; doi:10.3390/ijms25116241)
Supplement: Supplementary file 1 [file ijms-25-06241-s001.zip › Supplementary legends.docx]

Figure S1: The phylogenetic tree of WRKY complete amino acid sequences in grape with expanded clusters;

Table S1: The identified WRKY genes and chromosome location in different grape cultivars and assemblies;

Figure S2: The WRKY signature and zinc-finger motif in WRKY TFs in grape.
