# Supplementary material for: Genome-Wide Identification, Characterisation, and Evolution of the Transcription Factor WRKY in Grapevine (Vitis vinifera): New View and Update
Source: Int J Mol Sci. 2024 Jun 5;25(11):6241. doi: 10.3390/ijms25116241 (PMC11172563; doi:10.3390/ijms25116241)

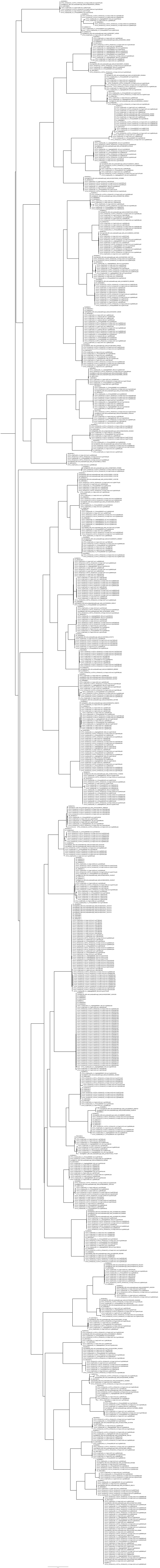

Supplement: Supplementary file 1 [file Supplementary Figure S1.pdf › Supplementary Figure S1.pdf]
